# A psittacosaurid-like basal neoceratopsian from the Upper Cretaceous of central China and its implications for basal ceratopsian evolution

**DOI:** 10.1038/srep14190

**Published:** 2015-09-21

**Authors:** Wenjie Zheng, Xingsheng Jin, Xing Xu

**Affiliations:** 1 Key Laboratory of Vertebrate Evolution and Human Origins of Chinese Academy of Sciences, Institute of Vertebrate Paleontology and Paleoanthropology, Chinese Academy of Sciences, Beijing 100044, People’s Republic of China; 2 University of Chinese Academy of Sciences, Beijing 100049, People’s Republic of China; 3 Zhejiang Museum of Natural History, Hangzhou, Zhejiang 310014, People’s Republic of China

## Abstract

Psittacosauridae (parrot-beaked dinosaurs) represents the first major radiation of ceratopsians (horned dinosaurs). However, psittacosaurids are divergent from the general morphology found in other ceratopsians, and this has resulted in their uncertain systematic position among ceratopsians. Here we describe a new basal neoceratopsian dinosaur, *Mosaiceratops azumai* gen. et sp. nov. based on a partial semi-articulated skeleton recovered from the Upper Cretaceous Xiaguan Formation of Neixiang County, Henan Province, China. Although our phylogenetic analysis supports this taxon as the most basal neoceratopsian, *Mosaiceratops* exhibits many features previously considered unique to the Psittacosauridae among the basal Ceratopsia. These include a relatively highly positioned external naris, a proportionally large premaxilla, the nasal extending ventral to the external naris, slender postorbital and temporal bars, a large notch between the basal tubera, and the edentulous premaxilla. Thus, the discovery of *Mosaiceratops* reduces the morphological disparity between the Psittacosauridae and other basal ceratopsians. Character optimization suggests that basal neoceratopsians have re-evolved premaxillary teeth; a major reversal previously unknown in any dinosaur clade. The new specimen also highlights the mosaic nature of evolution among early ceratopsians and supports the phylogenetic hypothesis that the Psittacosauridae is a relatively derived clade, rather than the most basal group of the Ceratopsia.

Ceratopsians are a group of ornithischian dinosaurs characterized by a number of features including a large head, a skull with narrow beak and flaring jugals, a rostral bone, a bony frill, and other characters^1^,^2^,^3^. Among the known ceratopsians, Psittacosauridae is the most unusual clade because it differs from other ceratopsians in many features, such as its bipedal posture, high and short snout, highly positioned external naris, and reduced lateral manual digits^4^,^5^. The significant morphological differences between the psittacosaurids and other ceratopsians hampered the recognition of the ceratopsian affinity of the Psittacosauridae when they were first discovered^6^,^7^,^8^. The recent discovery of Jurassic ceratopsians has helped to reduce the morphological gap between the Psittacosauridae and other ceratopsians^9^,^10^,^11^,^12^. However, significant morphological differences still remain. As a result, the systematic position of the Psittacosauridae remains controversial with some studies considering them as the most basal ceratopsian clade^5^,^13^,^14^, and others placing the group as intermediate between Jurassic forms such as Chaoyangsauridae and other Cretaceous taxa^2^,^12^.

Here we report a new basal neoceratopsian based on a specimen recovered from the Upper Cretaceous Xiaguan Formation of Neixiang County, Henan Province, China. The locality is on the west bank of Tuanhe River lies in the center of the Xiaguan-Gaoqiu Basin, one of several Cretaceous terrestrial basins in southwestern Henan Province. Xiaguan-Gaoqiu Basin previously has yielded a large number of fossil eggs and two dinosaur taxa, the basal hadrosauroid *Nanyangosaurus*^15^ and the titanosauriform *Baotianmansaurus*^16^. The fossil-bearing horizon in the Xiaguan-Gaoqiu Basin has been assigned variously to the Sangping^15^, Gaogou^16^, and Majiachun formations^17^. Despite those previous assessments, the horizon is now placed in the Xiaguan Formation^17^,^18^. The horizon lacks radiometric dates, but is believed to fall between the early-middle Turonian and the middle Campanian of the Late Cretaceous, based on the plant and invertebrate fossils^17^. This new specimen displays a mosaic combination of features, some of which were previously considered diagnostic of a certain ceratopsian clade, notably the Psittacosauridae. This specimen represents a significant discovery for understanding the early evolution of ceratopsian dinosaurs. The new information provided by this specimen reshapes our hypotheses of the evolution of several features in ceratopsian evolution.

## Results

### Systematic palaeontology

Dinosauria Owen, 1842

Ornithischia Seeley, 1887

Ceratopsia Marsh, 1890

Neoceratopsia Sereno, 1986

*Mosaiceratops azumai* gen. et sp. nov.

#### Etymology

The generic name *Mosaiceratops* (“mosaic ceratopsian”) is a contraction of the Latin terms “mosaicus” and “ceratops” in reference to the specimen’s unique (mosaic) combination of characters that were previously considered diagnostic of basal ceratopsians, psittacosaurids, or basal neoceratopsians. The specific name honors Dr. Yoichi Azuma from Fukui Prefectural Dinosaur Museum, who co-organized and participated in several dinosaur expeditions in China. One of those expeditions led to the discovery of the basal neoceratopsian *Archaeoceratops*^19^.

#### Holotype

**Figure 1:**
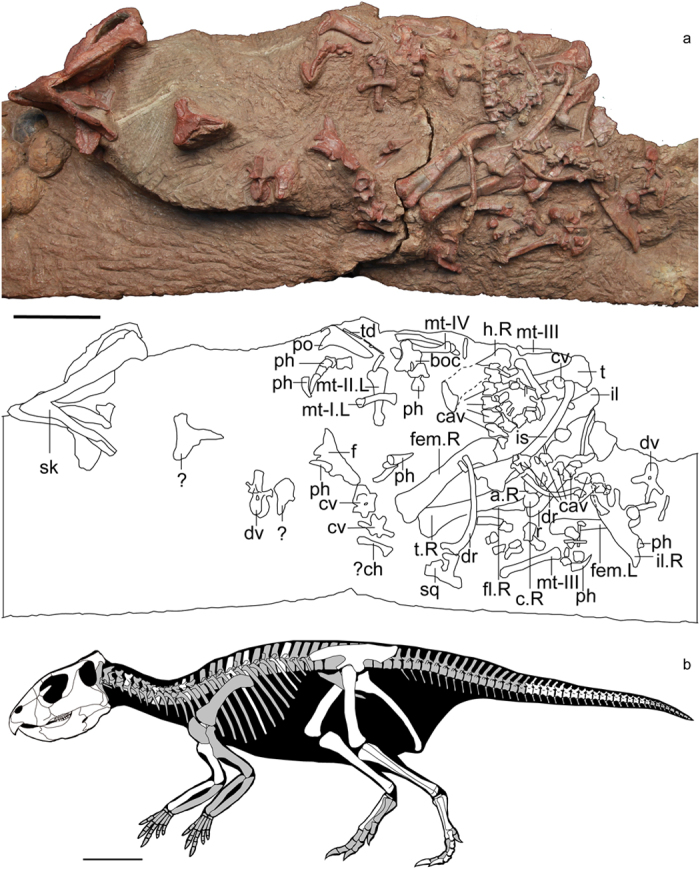
Holotype and skeletal reconstruction of *Mosaiceratops azumai*, gen. et sp. nov (ZMNH M8856). (**a**) photograph and line drawing of ZMNH M8856; (**b**) skeletal reconstruction showing preserved elements in white. Scale bar 10 cm. Abbreviations: a, astragalus; boc, basioccipital; c, calcaneum; cav, caudal vertebra; ch, chevron; cv, cervical vertebra; dr, dorsal rib; dv, dorsal vertebra; f, frontal; fem, femur; fl, fibula; h, humerus; il, ilium; is, ischium; L, left; mt, metatarsal; ph, phalanx/phalanges; po, postorbital; R, right; sk, skull; sq, squamosal; t, tibia; td, tendon; ?, undiagnostic remains.

**Figure 2:**
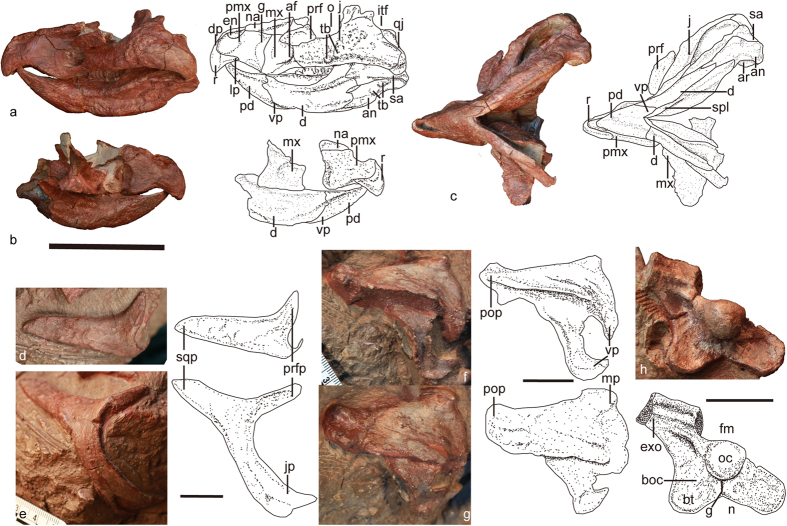
Cranial remains of *Mosaiceratops azumai*, gen. et sp. nov. Photograph and interpretive drawing of skull in left lateral (**a**), right lateral (**b**) and ventral (**c**) views; right postorbital in dorsal (**d**) and anterolateral (**e**) views; left squamosal in lateral (**f**) and laterodorsal (**g**) views; partial braincase in occipital (**h**) view. Scale bars in a–c, 5 cm, in d–h, 2 cm. Abbreviations: af, antorbital fossa; an, angular; ar, articular; boc, basioccipital; bt, basioccipital tubera; d, dentary; dp, dorsal process of rostral; en, external naris; exo, exoccipital; fm, foramen magnum; g, groove; itf, infratemporal fenestra; j, jugal; jp, jugal process; lp, lateral process; mp, medial process; mx, maxilla; n, notch; na, nasal; o, orbit; oc, occipital condyle; pd, predentary; pmx, premaxilla; pop, postorbital process; prf, prefrontal; prfp, prefrontal process; qj, quadratojugal; r, rostral bone; sa, surangular; spl, splenial; sqp, squamosal process; tb, tubercle; vp, ventral process.

ZMNH M8856 (housed in the collections of the Zhejiang Museum of Natural History, Hangzhou, China) (Figs 1 and 2) is composed of a partial skull and associated partial postcranial skeleton that includes three cervical vertebrae, three dorsal vertebrae, dorsal ribs, 18 caudal vertebrae, chevrons, the right humerus, the radius, both ilia, the left ischium, both femora, both tibiae, the left fibula, the left astragalus, the left calcaneum, metatarsals, phalanges, and some undiagnostic remains.

#### Locality and horizon

Upper Cretaceous (lower-middle Turonian—middle Campanian), Xiaguan Formation, Neixiang County, Henan Province, China.

#### Diagnosis

Small basal neoceratopsian with the following autapomorphies: curved groove anterior to the contact between the premaxilla and maxilla; elongate anterior process of jugal with sub-parallel dorsal and ventral edges; two tubercles developed in the ventral border infraorbital ramus; and the distal end of the dorsal ilium edge dorsoposteriorly directed. *Mosaiceratops* also differs from other basal neoceratopsians in the following features: edentulous premaxilla; premaxilla slightly larger than maxilla in lateral view; nasal extended ventral to the external naris; two tubercles close to the ventral border of the infraorbital ramus of the jugal; an inverted T-shape jugal in lateral view; a T-shape postorbital with slender and elongate jugal and squamosal processes; medial process of squamosal anteromedially directed; anterior process of the squamosal simple, rather than bifid; and basal tubera separated by a deep middle notch.

### Description and comparisons

The skull has the characteristic ceratopsian triangular morphology in dorsal view, with a narrow beak and strongly flaring jugal (Fig. 2c). The external naris sits relatively high with its ventral border slightly higher than the ventral edge of the orbit. Although incomplete, the external naris seems elongate, as characteristic of other basal neoceratopsians. In contrast whereas the naris is circular in *Psittacosaurus*^5^. The antorbital fossa is subtriangular in outline with sharp ventral and posterior rims as in *Archaeoceratops*^20^, *Leptoceratops*, and *Protoceratops*^14^, and the antorbital fossa is absent in *Psittacosaurus*^5^. The ventral border of the infratemporal fenestra sits lower than the ventral border of the orbit, similar to other neoceratopsians such as *Liaoceratops*, and *Archaeoceratops,* but contrasts with that of *Psittacosaurus*^5^.

The rostral extends anteriorly beyond the anterior tip of the lower jaw and curves strongly ventrally in lateral view (Fig. 2a). It appears round and unkeeled (Fig. 2c), similar to *Chaoyangsaurus*^10^, *Psittacosaurus*^5^, *Liaoceratops*^12^, and *Yamaceratops*^21^. In contrast, the rostral is keeled in most neoceratopsians, e.g. *Archaeoceratops*^20^. As in other neoceratopsians, the rostral bears lateral processes that extend posteriorly along the ventral margin of the premaxilla^21^.

The premaxilla appears subrectangular with a deeper posterior portion more closely resembling *Archaeoceratops* than those of other basal neoceratopsians (Fig. 2a)^20^. However, it is proportionally longer and lower than *Archaeoceratops*. As in psittacosaurids, the premaxilla is larger than the maxilla in lateral view. This differs from the condition in other ceratopsians such as *Liaoceratops*^12^, *Archaeoceratops*^19^, and *Auroraceratops*^22^, in which the premaxilla is smaller. The premaxillae and rostral bones form sharp, edentulous cutting margins (Fig. 2a–c), a feature similar to *Psittacosaurus* but not other basal ceratopsians^5^. A curved groove lies anterior to the premaxillary−maxillary suture, with its dorsal and ventral ends bending toward the posterior end of the premaxilla dorsally and ventrally, respectively. The maxilla widens posteriorly beneath the antorbital fossa (Fig. 2a). The dentigerous margin of the maxilla is inset more strongly than that of basal ceratopsians, such as *Yinlong*^9^ and *Chaoyangsaurus*^10^. More than ten teeth are present in the left maxilla, in addition to two disarticulated teeth preserved adjacent to the maxilla.

The external surface of the nasal has a roughened texture with no indication of a nasal horn, similar to other basal ceratopsians^5^. The nasal extends anteroventrally below the external naris, as in *Psittacosaurus*^5^.

The shape of the jugal resembles an inverted T in lateral view (Fig. 2a), similar to basal ceratopsians and *Psittacosaurus*. The infraorbital ramus of the jugal is shallow, elongate, and its dorsal and ventral edges are sub-parallel to each other in lateral view (Fig. 2a). In other basal neoceratopsians, e.g. *Archaeoceratops*^20^ and *Yamaceratops*^21^, the infraorbital ramus is deep near the base of the dorsal process and tapers anteriorly with a sub-triangular morphology in lateral view. The lateral surface bears a distinct bumpy ornamentation. There are two tubercles close to the ventral border of the infraorbital ramus that are not present in other neoceratopsians (Fig. 2a). The jugal bears a low horn that lacks a separate epijugal, as in *Liaoceratops*^12^. As in the neoceratopsians more derived than *Liaoceratops*, the quadratojugal appears more prominent in occipital than lateral view^21^.

The triradiate postorbital retains elongate jugal and squamosal processes and a shortened prefrontal process (Fig. 2d,e), as in *Psittacosaurus*. In contrast, the postorbital in basal neoceratopsians is a triangular plate of bone, the jugal process is shorter, and the squamosal process is more stout^5^. The postorbital process of the squamosal does not bifurcate (Fig. 2f,g), like the condition in *Yinlong*^9^ and *Psittacosaurus*^5^. However, the postorbital process is bifid in the other basal neoceratopsians, such as *Liaoceratops*, *Archaeoceratops*, and the leptoceratopsids^21^. Although broken, the medial ramus is anteromedially directed (Fig. 2g), similar to that in *Yinlong*^9^ and *Psittacosaurus*^5^.

The foramen magnum is slightly larger than the occipital condyle, in contrast to the condition in *Montanoceratops* (Fig. 2h)^23^. The occipital condyle shares the spherical shape and distinct neck with other neoceratopsians^5^. The basioccipital flares laterally below the condyle to form the large basal tubera that is characteristic of ceratopsians^23^. A shallow groove occurs in the midline of the tubera, and is also present in *Montanoceratops*^23^. In contrast, a longitudinal ridge is present below the condyle in *Yinlong*^9^ and other basal neoceratopsians, such as *Liaoceratops*^24^ and *Archaeoceratops*^19^. The basal tubera are widely separated by a middle notch, similar to *Psittacosaurus*^25^,^26^,^27^,^28^. This notch is absent or incipient in other neoceratopsians including *Archaeoceratops*^20^ and *Auroraceratops*^25^.

The mandibular ramus is shallow as in other basal neoceratopsians (Fig. 2a). In ventral view, the mandibular ramus expands posteriorly (Fig. 2c), resembling other basal neoceratopsians. The mandibular ramus appears nearly straight in dorsal view in *Yinlong*, *Psittacosaurus*, and *Chaoyangsaurus*^29^. The ratio of the length posterior to the apex of the coronoid process to total mandibular length is approximately 0.35, similar to other basal neoceratopsians. This ratio is smaller than that in more basal ceratopsians and *Psittacosaurus*^29^.

The relatively long predentary possesses a strongly upturned and acutely pointed anterior tip that fits inside the upper beak (Fig. 2a). That feature is present in most other neoceratopsians including *Archaeoceratops*^20^ and *Auroraceratops*^25^. In contrast, the predentary is short and blunt in the basal neoceratopsian *Liaoceratops*^24^ and the more basal ceratopsians *Yinlong*^9^, *Chaoyangsaurus*^10^, and *Psittacosaurus*^5^. The dorsal edges are beveled, a feature present in many ceratopsians. In ventral view, the predentary displays a flattened ventromedial process that bifurcates posteriorly (Fig. 2c). The anteroventral surface accommodates a sharp keel at the midline. The dentary bears an everted flange extending laterally (Fig. 2a), as in *Liaoceratops*^12^. This feature is well developed and directed ventrally in *Psittacosaurus*^29^. The coronoid process sits low but still relatively higher than that of basal ceratopsians and *Psittacosaurus*. The process is well developed and separated from the tooth row by a wide sulcus, unlike *Chaoyangsaurus*, *Psittacosaurus*, and *Liaoceratops*. There are approximately eleven tooth positions in the dentary.

The splenial is restricted to the medial margin of the mandible, with little ventral exposure. The anterior end of the splenial reaches the posteroventral end of the mandibular symphysis (bifurcate point of the predentary). That condition is similar to *Liaoceratops*^30^ and *Leptoceratops*^29^, but contrasts with the state in *Archaeoceratops*^29^.

The surangular is shorter than the dentary (Fig. 2a), and their dorsal and lateral surfaces are almost perpendicular to each other. The surangular forms part of the glenoid fossa lateral to the articular and comprises more than half of the lateral cotyle of the glenoid, a condition common in basal neoceratopsians^31^. The retroarticular process is short. The medial and lateral cotyles of the glenoid are well separated by a ridge. The elliptical and gently dorsally concave, elevated glenoid surface contrasts with the flat articular surface of the mandibular condyle in *Psittacosaurus*^5^,^32^. Two small tubercles are present near the posterior end of the angular. The larger one occurs on the suture with the surangular, and the smaller one occurs anteroventrally to the previous tubercle. A weak ridge is developed along these two tubercles and is confluent with the dentary flange.

The maxillary tooth crowns have a low ridge distal to the middle line labially, similar to *Archaeoceratops*^33^. The primary ridge is narrow and the width remains constant, like that in *Psittacosaurus lujiatunensis*, *Psittacosaurus sinensis*, and *Archaeoceratops*^33^. In contrast, the primary ridges taper toward apex in other species of *Psittacosaurus*, *Yinlong*, and *Auroraceratops*^33^. Two or three subsidiary ridges are present medial to the prominent primary ridge that are parallel to each other, but oblique to the primary ridge (as in *Archaeoceratops*^33^). The teeth are single rooted as in other non-ceratopsid ceratopsians like *Chaoyangsaurus*, *Psittacosaurus*^33^, and *Leptoceratops*^34^,^35^. The dentary tooth crowns have a primary ridge lingually with three to four distal secondary ridges. The primary ridge is narrow like other basal neoceratopsians (*Archaeoceratops* and *Auroraceratops*^33^). In contrast, the primary ridge is more prominent and bulbous in *Psittacosaurus*^5^,^33^. In one dentary tooth, distal secondary ridges are parallel with the primary ridge, but in other dentary teeth, the distal secondary ridges are directed obliquely to the primary ridge.

The neurocentral suture is closed in all three preserved cervical vertebrae. The anterior cervical displays a well-developed ventral keel that becomes weaker in the posterior cervical. In the caudal vertebrae, the transverse processes are not fully fused with the centrum. The longest neural spines are approximately 1.5 times that of the corresponding centrum length.

As in other basal neoceratopsians, the humeral head inclines medially in posterior view. It is hemispherical in proximal view and supported by a thickened tubercle that projects to the posterior surface. In contrast, the head is poorly defined in *Psittacosaurus*^5^. The relatively long deltopectoral crest broadly expands and faces anteriorly. The crest is limited to the proximal half of shaft in *Mosaiceratops*, whereas it extends to the distal half in ceratopsids^35^. As in other neoceratopsians^31^,^34^,^36^, the medial condyle extends slightly further than the lateral condyle.

The gently arched dorsal margin of the ilium has its apex over the acetabulum, and the dorsal margin of the postacetabular process curves slightly dorsally at the posterior end (Fig. 1a). The postacetabular process in basal neoceratopsians normally curves ventrally towards the posterior end, as in *Archaeoceratops*^20^, *Yamaceratops*^21^, and *Auroraceratops*^31^. The narrow pubic peduncle projects anteroventrally, and the much more robust ischial peduncle contains a large convex antitrochanter. The ischial shaft appears oval in cross section like *Auroraceratops*, but not laterally compressed as in *Psittacosaurus lujiatunensis*, *Leptoceratops*, *Montanoceratops*, and *Protoceratops*^31^. The distal end of the ischium downturns slightly and swells with a rugose surface, similar to other basal neoceratopsians such as *Auroraceratops*^37^ and *Protoceratops*^36^. However, the ischium is robust and decurved more strongly in ceratopsids^35^.

The femur gently bows anteriorly in lateral view (Fig. 1a). The fourth trochanter is large and only slightly pendent, as in other non-ceratopsid ceratopsians^5^,^35^. The shaft of the tibia is straight and longer than the femur (Fig. 1a), and comparable to other basal ceratopsians. The opposite condition is present in ceratopsids^35^,^38^. The foot is gracile and elongate with a constricted metatarsus and elongate phalanges (Fig. 1a). The ungual phalanges are slender and sharply pointed, as in other basal ceratopsians^14^ such as *Archaeoceratops*^20^ and *Leptoceratops*^34^.

## Methods

### Phylogenetic analysis

In order to determine the systematic position of *Mosaiceratops azumai*, we conducted a phylogenetic analysis with a dataset modified from a recently published dataset for ceratopsian phylogeny^39^. The major modifications include 12 new characters, four additional taxa [*Mosaiceratops*, *Auroraceratops*^22^,^31^, *Zhuchengceratops*^40^, and *Aquilops*^41^], and the revision of some character coding (see Supplementary Information). The modified matrix is comprised of 159 characters and 26 taxa.

**Figure 3:**
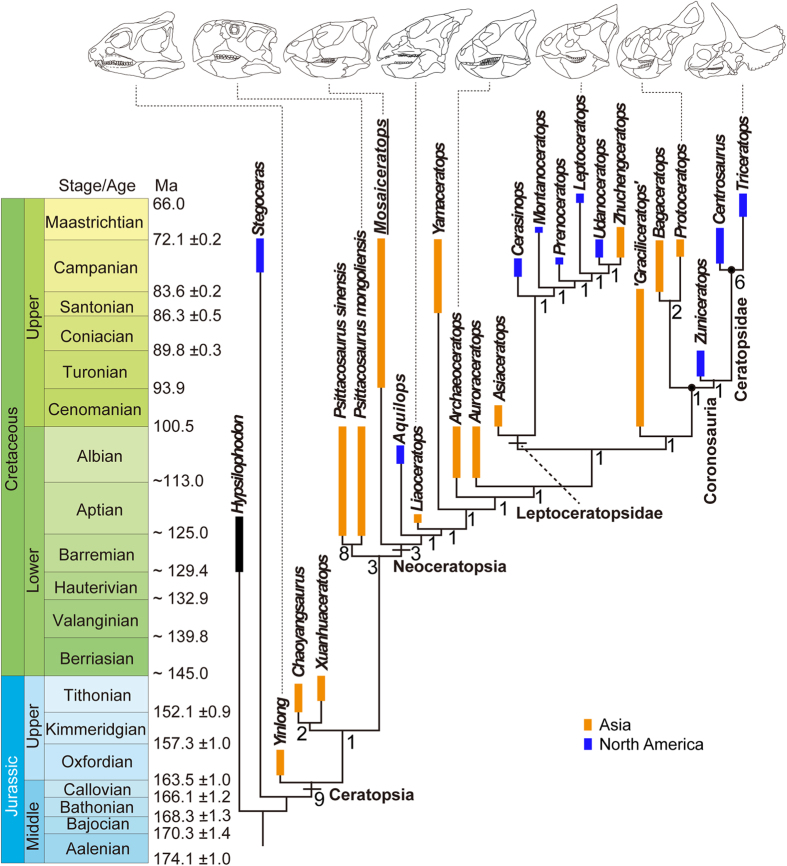
Temporal calibration of the single most parsimonious tree produced by phylogenetic analysis. Numbers beneath nodes are Bremer support values. The geologic numerical ages and coloring follow International Chronostratigraphic Chart 2014/10^48^.

The analysis was carried out using a traditional search with the tree bisection reconnection algorithm in TNT version 1.1^42^. The parameters were left at their default settings with the following exception: maximum trees in memory set to 10000, 1000 replicates were used with 10 trees saved per replication. The analysis resulted in one parsimonious tree (tree length = 339 steps, CI = 0.54, and RI = 0.71). *Mosaiceratops* is recovered as the most basal neoceratopsian. The Jurassic forms, such as *Yinlong* and the Chaoyangsauridae, are placed at the base of the Ceratopsia, and the Psittacosauridae are positioned intermediate between the Jurassic forms and the Neoceratopsia (Fig. 3).

### Stratigraphic fit analysis

Measures of stratigraphic fit to phylogeny were analyzed by calculating modified Manhattan stratigraphic measure (MSM*) in TNT version 1.1^42^,^43^,^44^. Temporal calibrations used for fit analysis are provided in Supplementary Information.

## Discussion

*Mosaiceratops* clearly is a member of the Ceratopsia based on the presence of a rostral bone, the narial fossa being separated by a flat margin from the ventral margin of the premaxilla, the laterally projected and crested jugal, and the predentary with a wide ventral process. Furthermore, this new species can be placed in the Neoceratopsia based on the presence of several derived features known only in neoceratopsians: a rostral with a well-developed lateral process and a strongly ventrally curved ventral edge; a quadratojugal more prominent in occipital view than lateral view; a spherical occipital condyle; a predentary with a beveled dorsal margin; a predentary that is relatively long and keeled, and has an acutely pointed and strongly upturned anterior tip; shortened post-dentary mandibular elements; a reduced retroarticular process; and the hemispherical humeral head extending onto the posterior surface of the humerus.

*Mosaiceratops* possesses several plesiomorphic features that are previously unknown in other basal neoceratopsians. For example, the postorbital is T-shape with an elongation of the jugal and squamosal processes (similar to *Yinlong* and *Psittacosaurus*). However, the postorbital is a triangular plate of bone with stout jugal and squamosal processes in other basal neoceratopsians. The jugal is an inverted T-shape in lateral view, like that in *Yinlong* and *Psittacosaurus*. However, the jugal appears sub-triangular in other basal neoceratopsians. The simple postorbital process of the squamosal in *Mosaiceratops* appears similar to *Yinlong* and *Psittacosaurus*, and the postorbital inserts into the bifid anterior end of the squamosal in other basal neoceratopsians. The medial process of the squamosal angles anteromedially, as in *Yinlong* and *Psittacosaurus*. However, the medial process of the squamosal is directed posteromedially to form the lateral corner of the frill.

*Mosaiceratops* possesses several features previously only known in the Psittacosauridae among basal ceratopsians and other basal neoceratopsians. As in the Psittacosauridae, the external naris is positioned relatively high, the nasal extends ventral to the external naris and reaches the rostral, and there is a large notch between the basal tubera. The most striking feature is the edentulous premaxilla in *Mosaiceratops*. Although derived ceratopsians such as most Leptoceratopsids (*Montanoceratops*, *Prenoceratops*, *Leptoceratops*, and *Udanoceratops*)^39^, *Bagaceratops*^6^, and Ceratopsids^35^ lack premaxillary teeth, all other basal ceratopsians have premaxillary teeth. For example, *Yinlong* has three premaxillary teeth^9^, *Archaeoceratops* has three^19^, and *Protoceratops* has two^36^. Among the basal ceratopsians and basal neoceratopsians, only the psittacosaurids lack premaxillary teeth. *Mosaiceratops* represents the first known non-psittacosaurid taxon among basal ceratopsians that lacks premaxillary teeth. Character optimization indicates that the absence of premaxillary teeth is a diagnostic feature for a clade composed of the Psittacosauridae and the Neoceratopsia, and the occurrence of premaxillary teeth in other basal neoceratopsians, more derived than *Mosaiceratops,* represents a reversal to the more primitive condition present in basal ceratopsians. The potential reappearance of premaxillary teeth among more derived neoceratopsians would be a major character reversal previously unknown in any dinosaur clade, and the absence of premaxillary teeth in *Mosaiceratops* helps to indicate another example of the occurrence of major homoplasy in dinosaur evolution. One other example is the re-evolving of a robust, functional hallux in derived therizinosaurs^45^. Major reversals also are known in several other vertebrate taxa, for example, the re-evolution of lost digits in lizard *Bachia*^46^ and re-evolution of lost mandibular teeth in frogs^47^. However, the recovered systematic position of *Mosaiceratops* as the most basal neoceratopsian is not strongly supported (Bremer index of 3) in our phylogenetic analysis, and the possibility that *Mosaiceratops* represents the sister taxon to *Psittacosaurus* exists. In that less parsimonious case, the loss of premaxillary teeth would characterize a much less inclusive clade, and no major reversal would have occurred in ceratopsian evolution.

It should be noted that some features in *Mosaiceratops* display an intermediate condition between those of psittacosaurids and neoceratopsians. For example, the premaxilla is larger than the maxilla in lateral view, but not proportionally as large as in psittacosaurids. In the latter, the premaxilla occupies the majority of the lateral surface of the snout. In other neoceratopsians, the maxilla overshadows the premaxilla in size in lateral view. The external naris sits relatively high on the face, its ventral border slightly higher than the ventral edge of the orbit. The external naris sits high in psittacosaurids, and lies just below the upper portion of orbit. However, in other neoceratopsians, the external naris is positioned lower than the orbit. In our specimen, the basal tubera are separated by a deep middle notch. In psittacosaurids, the basal tubera also are separated by a deep middle notch, but the notch is deeper and narrower. In some neoceratopsians, the ventral border of basal tubera is slightly concave or almost flat. *Mosaiceratops* exhibits a laterally directed jaw flange that also is present in *Liaoceratops*. However, the jaw flange is well developed in psittacosaurids and directed ventrally, and it is absent among other neoceratopsians^12^.

The discovery of *Mosaiceratops* highlights the mosaic evolution of basal ceratopsians that was also demonstrated by the previous discovery of *Liaoceratops*^12^*. Mosaiceratops* combines several derived features seen across different ceratopsian clades while combining those traits with many psittacosaurid-like features. These mingled characters in this basal neoceratopsian reduce the morphologic gap between the Psittacosauridae and other basal ceratopsians, but suggests a couple of evolutionary scenarios. If *Mosaiceratops* is the most basal neoceratopsian, then certain features (discussed above) are plesiomorphic for neoceratopsians rather than being derived solely for psittacosaurids and others have a distinctly homoplastic distribution. On the other hand, if *Mosaiceratops* is actually a basal psittacosaurid (the less parsimonious hypothesis), then it would suggest that psittacosaurids evolved from a much more neoceratopsian-like ancestor than currently thought.

The Early Cretaceous Psittacosauridae have been considered intermediate in phylogenetic position between the Jurassic ceratopsians, such as *Yinlong* and the Chaoyangsauridae, and all other Cretaceous ceratopsians (i.e., the Neoceratopsia)^9^,^21^,^39^. However, a few studies suggest that the Psittacosauridae are more basal than the Chaoyangsauridae in ceratopsian phylogeny^5^,^13^,^14^. Our phylogenetic analysis incorporating new information from *Mosaiceratops* strongly supports the hypothesis that the Psittacosauridae are more derived than Chaoyangsauridae. The (Chaoyangsauridae + (Psittacosauridae + Neoceratopsia)) phylogenetic hypothesis is more consistent with the stratigraphic record than the (Psittacosauridae + (Chaoyangsauridae + Neoceratopsia)) hypothesis, indicated by our comparative stratigraphic fit indexes (MSM* values are 0.20 and 0.19 respectively). However, it should be pointed out that in general the calculated MSM* value is low and many relatively long ghost lineages exist (including the lineage leading to *Mosaiceratops*). Those significant gaps in the fossil record suggest great potential for future paleontological discoveries that hopefully will resolve some of the evolutionary history of the major features of the ceratopsian skeleton.

## Additional Information

**How to cite this article**: Zheng, W. *et al.* A psittacosaurid-like basal neoceratopsian from the Upper Cretaceous of central China and its implications for basal ceratopsian evolution. *Sci. Rep.* **5**, 14190; doi: 10.1038/srep14190 (2015).

## Supplementary Material

Supplementary Information

## References

[b1] Sereno P. C. Phylogeny of the bird-hipped dinosaurs (Order Ornithischia). National Geographic Research 2, 234–256 (1986).

[b2] Makovicky P. J. in The complete dinosaur (eds Brett-Surman Michael K, Holtz Thomas R, Farlow James O & Walters Bob ) Ch. 25, 527–550 (Indiana University Press, 2012).

[b3] Dodson P. The horned dinoaurs. 346 (Princeton University Press, 1996).

[b4] Sereno P. C. in The Dinosauria (eds Weishampel D. B., Dodson P. & Osmolska H. ) 579–592 (University of California Press, 1990).

[b5] You H.-L. & Dodson P. in The Dinosauria (eds Weishampel David B., Dodson Peter & Osmólska Halszka ) Ch. 22, 478–493 (The University of California Press, 2004).

[b6] Maryańska T. & Osmólska H. Protoceratopsidae (Dinosauria) of Asia. Palaeontologica Polonica 33, 133–181 (1975).

[b7] Coombs W. P. J. Juvenile specimens of the ornithischian dinosaur *Psittacosaurus mongoliensis*. SO - Palaeontology (Oxford). 25(1). 1982, 89–108 (1982).

[b8] Osborn H. F. Two Lower Cretaceous dinosaurs of Mongolia. American Museum Novitates 95, 1–10 (1923).

[b9] Xu X., Forster C. A., Clark J. M. & Mo J. A basal ceratopsian with transitional features from the Late Jurassic of northwestern China. Proceedings of the Royal Society B: Biological Sciences 273, 2135–2140, 10.1098/rspb.2006.3566 (2006). 16901832 PMC1635516

[b10] Zhao X.-J., Cheng Z.-W. & Xu X. The earliest ceratopsian from the Tuchengzi Formation of Liaoning, China. Journal of Vertebrate Paleontology 19, 681–691 (1999).

[b11] Zhao X., Cheng Z., Xu X. & Makovicky P. J. A new ceratopsian from the Upper Jurassic Houcheng Formation of Hebei, China. Acta Geologica Sinica - English Edition 80, 467–473, 10.1111/j.1755-6724.2006.tb00265.x (2006).

[b12] Xu X., Makovicky P. J., Wang X.-l., Norell M. A. & You H.-l. A ceratopsian dinosaur from China and the early evolution of Ceratopsia. Nature 416, 314–317, 10.1038/416314a (2002). 11907575

[b13] Sereno P. C. The origin and evolution of dinosaurs. Annual Review of Earth and Planetary Sciences 25, 435–489, 10.1146/annurev.earth.25.1.435 (1997).

[b14] Sereno P. C. in The Age of Dinosaurs in Russia and Mongolia (eds Benton Michael J., Shishkin Mikhail A., Unwin David M. & Kurochkin Evgenii N. ) Ch. 25, 480–516 (Cambridge University Press, 2000).

[b15] Xu X. *et al.* A new iguanodontian from Sangping Formation of Neixiang, Henan and its stratigraphical implication. Vertebrata PalAsiatica 38, 176–191 (2000).

[b16] Zhang X. *et al.* A new sauropod dinosaur from the Late Cretaceous Gaogou Formation of Nanyang, Henan Province. Acta Geologica Sinica - English Edition 83, 212–221, 10.1111/j.1755-6724.2009.00032.x (2009).

[b17] Wang D. *et al.* Discovery of invertebrate zoolite in the Xiaguan Formation of Xiaguan-Gaoqiu Basin, Henan, China, and its importance for stratigraphic subdivision comparison. Acta Geologica Sinica 87, 1049–1058 (2013).

[b18] Zhou S.-Q., Zhu G.-B. & Feng Z.-J. Xiaguan Formation and its era of Xiaguan-Gaogiu Basin, in Neixiang county, Henan Province. Resources Survey & Environment 24, 69–74 (2003).

[b19] Dong Z. & Azuma Y. in Sino-Japanese Silk Road Dinosaur Expedition (ed Dong Zhiming ) 68–89 (China Ocean Press, 1997).

[b20] You H.-L. & Dodson P. Redescription of neoceratopsian dinosaur *Archaeoceratops* and early evolution of Neoceratopsia. Acta Palaeontologica Polonica 48, 261–272 (2003).

[b21] Makovicky P. J. & Norell M. A. *Yamaceratops Dorngobiensis*, a new primitive ceratopsian (Dinosauria: Ornithischia) from the Cretaceous of Mongolia. American Museum Novitates. 1–42, 10.1206/0003-0082(2006)3530[1:YDANPC]2.0.CO;2 (2006).

[b22] You H., Li D., Lamanna M. C. & Dodson P. On a new genus of basal neoceratopsian dinosaur from the Early Cretaceous of Gansu Province, China. Acta Geologica Sinica 70, 593–597 (2005).

[b23] Makovicky P. J. in Mesozoic Vertebrate Life (eds Tanke Darren H. & Carpenter Kenneth ) 243–262 (Indiana University Press, 2001).

[b24] Han F. The Osteology of Yinlong downsi (Ornithischia: Ceratopsia) and the Phylogeny of the ornithischian dinosaurs Doctor of Science thesis, University of Chinese Academy of Sciences (2013).

[b25] Dodson P., You H.-L. & Tanoue K. in New perspectives on horned dinosaurs: The Royal Tyrrell Museum Ceratopsian Symposium (eds Ryan Michael J., Chinnery-Allgeier Brenda J. & Eberth David A. ) 221–233 (Indiana University Press, 2010).

[b26] You H.-L., Tanoue K. & Dodson P. New data on cranial anatomy of the ceratopsian dinosaur *Psittacosaurus major*. Acta Palaeontologica Polonica 53, 183–196, 10.4202/app.2008.0202 (2008).

[b27] Sereno P. C. & Chao S. *Psittacosaurus xinjiangensis* (Ornithischia: Ceratopsia), a new psittacosaur from the Lower Cretaceous of northwestern China. Journal of Vertebrate Paleontology 8, 353–365, 10.1080/02724634.1988.10011724 (1988).

[b28] Sereno P. C. in New Perspectives on Horned Dinosaurs (eds Ryan Michael J., Chinnery-Allgeier Brenda J. & Eberth David A. ) Ch. 2, 21–58 (Indiana University Press, 2010).

[b29] Tanoue K., You H.-L. & Dodson P. in New perspectives on horned dinosaurs: The Royal Tyrrell Museum Ceratopsian Symposium (eds Ryan Michael J., Chinnery-Allgeier Brenda J. & Eberth David A. ) 234–250 (Indiana University Press, 2010).

[b30] You H., Tanoue K. & Dodson P. A New Specimen of *Liaoceratops yanzigouensis* (Dinosauria: Neoceratopsia) from the Early Cretaceous of Liaoning Province, China. Acta Geologica Sinica - English Edition 81, 898–904, 10.1111/j.1755-6724.2007.tb01011.x (2007).

[b31] Morschhauser E. M. The anatomy and phylogeny of Auroraceratops (Ornithischia: Ceratopsia) from the Yujingzi Basin of Gansu Province, China PhD thesis, University of Pennsylvania (2012).

[b32] Sereno P. C., Zhao X. & Tan L. A new psittacosaur from Inner Mongolia and the parrot-like structure and function of the psittacosaur skull. Proceedings of the Royal Society B: Biological Sciences 277, 199–209, 10.1098/rspb.2009.0691 (2010). 19535376 PMC2842669

[b33] Tanoue K., You H.-L. & Dodson P. Comparative anatomy of selected basal ceratopsian dentitions. Canadian Journal of Earth Sciences 46, 425–439, 10.1139/E09-030 (2009).

[b34] Brown B. *Leptoceratops*, a new genus of Ceratopsia from the Edmonton Cretaceous of Alberta. Bulletin American Museum of Natural History 33, 567–580 (1914).

[b35] Dodson P., Forster C. A. & Sampson S. D. in The Dinosauria (eds Weishampel David B., Dodson Peter & Osmólska Halszka ) Ch. 22, 494–513 (The University of California Press, 2004).

[b36] Brown B. & Schlaikjer E. M. The structure and relationships of *Protoceratops*. Annals of the New York Academy of Sciences 40, 133–266 (1940).

[b37] You H.-L., Morschhauser E. M., Dodson P. & Li D.-Q. *Auroraceratops* sp. (Dinosauria: Neoceratopsia) from the Early Cretaceous of the Mazongshan area in Northwestern China. Vertebrata PalAsiatica 50, 170–180 (2012).

[b38] Chinnery B. J. & Horner J. R. A new neoceratopsian dinosaur linking North American and Asian taxa. Journal of Vertebrate Paleontology 27, 625–641, 10.1671/0272-4634(2007)27[625:ANNDLN]2.0.CO;2 (2007).

[b39] Makovicky P. J. in New perspectives on horned dinosaurs: The Royal Tyrrell Museum Ceratopsian Symposium (eds Michael J.Ryan, Chinnery-Allgeier Brenda J. & Eberth David A. ) 68–82 (Indiana University Press, 2010).

[b40] Xu X., Wang K., Zhao X., Sullivan C. & Chen S. A new leptoceratopsid (Ornithischia: Ceratopsia) from the Upper Cretaceous of Shandong, China and its implications for neoceratopsian evolution. PLoS ONE 5, e13835, 10.1371/journal.pone.0013835 (2010). 21079798 PMC2973951

[b41] Farke A. A., Maxwell W. D., Cifelli R. L. & Wedel M. J. A ceratopsian dinosaur from the Lower Cretaceous of Western North America, and the Biogeography of Neoceratopsia. PLoS ONE 9, e112055, 10.1371/journal.pone.0112055 (2014). 25494182 PMC4262212

[b42] Goloboff P. A., Farris J. S. & Nixon K. C. TNT, a free program for phylogenetic analysis. Cladistics 24, 774–786, 10.1111/j.1096-0031.2008.00217.x (2008).

[b43] Siddall M. E. Forum. stratigraphic fit to phylogenies: a proposed solution. Cladistics 14, 201–208, 10.1006/clad.1998.0059 (1998). 34902929

[b44] Pol D. & Norell M. A. Comments on the Manhattan Stratigraphic Measure. Cladistics 17, 285–289, 10.1006/clad.2001.0166 (2001). 34911241

[b45] Xu X., Tang Z.-L. & Wang X.-L. A therizinosauroid dinosaur with integumentary structures from China. Nature 399, 350–354, 10.1038/20670 (1999).

[b46] Kohlsdorf T. & Wagner G. P. Evidence for the reversibility of digit loss: a phylogenetic study of limb evolution in *Bachia* (Gymnophthalmidae: Squamata). Evolution 60, 1896–1912, 10.1111/j.0014-3820.2006.tb00533.x (2006). 17089974

[b47] Wiens J. J. Re-evolution of lost mandibular teeth in frogs after more than 200 million years, and re-evaluating Dollo’s law. Evolution 65, 1283–1296, 10.1111/j.1558-5646.2011.01221.x (2011). 21521189

[b48] Cohen K. M., Finney S. C., Gibbard P. L. & Fan J.-X. The ICS International Chronostratigraphic Chart. Episodes 36, 199–204. (2013).

