# Supplementary material for: A psittacosaurid-like basal neoceratopsian from the Upper Cretaceous of central China and its implications for basal ceratopsian evolution
Source: Sci Rep. 2015 Sep 21;5:14190. doi: 10.1038/srep14190 (PMC4585677; doi:10.1038/srep14190)
Supplement: Supplementary Information [file srep14190-s1.pdf]

Supplementary Information

A psittacosaurid-like basal neoceratopsian from the Upper Cretaceous of central China and its implications for basal ceratopsian evolution

Wenjie Zheng[1, 2, 3], Xingsheng Jin[3] and Xing Xu[1]

[1]Key Laboratory of Vertebrate Evolution and Human Origins of Chinese Academy of Sciences, Institute of Vertebrate Paleontology and Paleoanthropology, Chinese Academy of Sciences, Beijing 100044, People's Republic of China
[2]University of Chinese Academy of Sciences, Beijing 100049, People's Republic of China
[3]Zhejiang Museum of Natural History, Hangzhou, Zhejiang 310014, People's Republic of China

Text S1. Revised and added character list used for phylogenetic analysis. Characters 1–133 are the same as Makovicky and Norell (2006: with character 15 modified), characters 134–147 are the same as Makovicky (2010). We added twelve characters (148-159) to the matrix.

15. Nares position close to buccal margin (0), or dorsal, away from buccal margin, but the ventral border of nares sits lower than the ventral border of the orbit (1), or the ventral border of nares sits slightly higher than the ventral border of orbit (2), or very far dorsal, level with upper part of orbit (3).

148. Basioccipital, a longitudinal ridge below condyle: present (0); or absent (1) (Xu et al., 2006:95; Han, 2013: 128).
149. External naris, shape: elliptical (0); round (1) (You and Dodson, 2003: 4; Morschhauser, 2012: 24).
150. Development of anteroventral chin (absent = 0, poorly developed = 1, well-developed = 2), (Ryan et al., 2012: 150)
151. Antorbital fossa, shape: subtriangular (0); oval (1) (Sereno, 2000: 21; You and Dodson, 2003: 7; Morschhauser, 2012: 41).
152. Jugal/epijugal crest, development; low (0); pronounced (1) (Sereno, 2000: 24; You and Dodson, 2003: 35; Morschhauser, 2012: 57).
153. Dentary depth: shallow, maximum dorsoventral height of dentary ramus less than 40% total dentary length (0); deep (1); very deep (2) (Morschhauser, 2012: 136).
154. Jaw articulation position relative to tooth row: below the tooth row level (0), at or above the tooth row (1) (Chinnery and Horner, 2007: 67; Morschhauser, 2012: 173)
155. The lateral aspect of premaxilla is smaller than maxilla (0), larger than maxilla (1), two times larger than maxilla (2).
156. Jugal dimension; longer than height (0), or higher than long (1).
157. Jugal shape; sub-triangular jugal (0), or sub-inverted-T-shaped (1) in lateral view
158. The jugal process of postorbital; short (0), elongate (1).
159. Basal tubera ventral notch weak or absent (0), present (1).

Text S2. Data matrix in TNT format.

xread

159 26

Hypsilophodon 0000?0--0000000000000001000000-0000000000000?0001100--
00000001?10000100000100010000000000000000000000000000000000000000000
000000000000-01000-?000000

Stegoceras 0000?0--000000000000000011000-0??010010?000000011100--
0000000?00000?00?000100100000000?0000000000000000000??0?0?10????00?0?0???0100011
?-0?0?0000-?100?-0000000

Archaeoceratops
1100011111011010000??01000111001?110??011?????11??10???11101?010011101?101010
??01?11110?10111000101???0?1000??0100?0?????????00??000001100000?0-0100010101000

Asiaceratops
?????1??1?????1?0???01??????????????????????????????????11?00??????????1101??0????
??01??????2-000?000?001??????????1????1???????????0??0??0???????????????0????

Bagaceratops
1100?1111101?01?01?0101000111101010101121100?111????1110111010010011111111110?0
11011111?111012-000201010011????1???????????????0??????000010?001?0-?10011110?000

Centrosaurus
1110011111011001121110001121111102110111121111110022 11?12110111011211021 1110?
010121011000?012-111201110111111212031011101011111111111000010000010-
1?10?1010??01

Leptoceratops
1100111111011012000011100011100110001101100??111?101000011?100110101111210001
0100110110101012-
00221101101101011101011101100000000000000000100000110 0?01111101000

Gracilliceratops
1100???????????????0??????1????????101111????????????100?1??????????????1?1??0??01
?111???1????0002010100????????1??1???1??????0??0000000?10?00000-???0?1????0?0

Montanoceratops
1????????1????1????010100?1?10011??011?11?????????0?0???112100?1010?1???1?10101
00111110??1012?0022110110?10111?11201110110?0000000000?00?10111??110??00111??0??

Protoceratops 110011111101001201001010001111010101011211001111 1?1211[0
1]0111010010011111111001011011111011101110002010100110111111201110110000100000010
00?10000000-1100111101000

Triceratops
1110111111001001121110001121111102110111121111110022101121101110112110211110?
0101210110001012-11120111011?11121203101110101111111111110000100 00010-
1?1011000??01

Udanoceratops
1??0111111?0??1?00?0?1????1110?????????11???????????????????????????1??21?0??0?00
1?011??01012-0022110110 11??????02011??1100??????0????000100??01????00?1210?0??

Zuniceratops
???????1?????????0???0????1???1??????????????????????????1?????????????11?1??0??0??
??????????-0?120101?01??????????0????????????1????0?00????01?0-?-?0?1100????
Yamaceratops
11??0110????1??????0101000101001010011011100000111010???111010?100111?1110101
0?001111110?1011000010001001 1??????0??1???????????????00000?10?00??0-01?00111??001
Prenoceratops
??0??1??1?00101?000??0100?1110011??011?11???????????0???11210??????11?12100010?
?011011???1012-0022110110?1???????????????????????????0?00?000000100?0111110?00?
Cerasinops
1????????????????????0????10??01???011?11?????????110100111100?1010?????1?0?????0
[1 2]1111???1?11?00210?0?1????0?1101[1 2]?1?1?1100?????1000000??10?00??0-
0??0?1?0??0?0
Zhuchengceratops
1????????????????????1???1?????????????????1111111??????????????????10??1100001011
100001?101???02211011011????010?????????????????????0???0?????101??0??21??1??
Auroraceratops
1101011111111011000100100010100101001111010001011111 0?1011?0100100001111101 0
10110110010101011000120101101101011112010010000000010000010001000?000-
000001010?000
Mosaiceratops 11?00110?1?11121000??010??100-
00000000?1?????????????????1?0???????1101?1?1110??01?0111101?12-
00010?0??0????????0??0????????????00000011??10??0?0-0100010110111
Liaoceratops ?10001101110101000001011001 00-
01010011001?00000111010110011010?10011?010100110000 1?010100101000001 00?10001????
???????????0???????????000110?000?0-?000010001000
Psinensis 110111001110113 2000??00000110-
00000000000?00????1?010010???????0???00001101000??01?000000??02-
00?0?000?000??0010?1000????10??????0?0000101100?000-?110-01?20111
Pmongoliensis 1101[0 1]100111011320001100[0
1]00110?0000000000000100011110100100100000100100001101[0 1]000?01?0000001002-
00000000000000000100000000111000000000000001011000000-0110-01120111
Chaoyangsaurus
110101001??00?120????0?1??010?????0????0????00001???????01?0???????1100?101001?
?00000010?1111000000000000000000000?????????????????????110??0?????0-0?????010????
Xuanhuaceratops
????????????0??11010?1?????
0?010?1111?00000000000000????0??????110???????0?0?110?????????-0?????????1??
Yinlong 11010100?0?01011000??00001000-01000000000000?00?1?00--
1100200??00?101001000000?00000001101?1100000000000?0000??0??00?0?????00100?0??011
01110000?0-?00?010000110
Aquilops ?1001111?1?1101?000??01000110-
0101?????????????????0?????????????????11?1110??10???????????????00000101010?01??????????
?????????????????0??0????00?????00010?0000?

;

proc /;
comments 0
;

Table S1. Measurements of ZMNH M8856. Asterisk indicates incomplete measurement due to damage or overlain by other remains.

		mm
skull	from the tip of the rostrum to the posterior end of mandible	155
Humerus (right)	proximodistal length	108
	proximal end, anteroposterior	15
	proximal end, mediolateral	38*
	distal end, anteroposterior	18
	distal end, mediolateral	22*
radius	proximodistal length	89
ulna	proximodistal length	
ilium right	length	147
ischium	length	167
	proximal width	44
femur right	length	137
	proximal end, anteroposterior	
	proximal end, mediolateral	
	distal end, anteroposterior	28
	distal end, mediolateral	32
femur left	length	128
tibia right	length (with ast)	145
tibia left	proximal end, anteroposterior	
astragalus right	anterior width	24
calcaneum right	length	12
mt-I	length	44
mt-II	length	66
mt-III	length	72
mt-IV	length	56
digit IV	phalanx 1 length	17
	phalanx 2 length	12
	phalanx 3 length	13
	phalanx 4 length	12
	phalanx 5 length	25

Table S2. Temporal calibrations used for fit analysis (Pol and Norell, 2001). All ages are given in Ma. The data mainly reference Farke et al. (2014), except the data of some Chinese ceratopsians, which indicated by the source column.

Taxon	Age	Lower Bound	Upper Bound	Midpoint	Date Assigned	Source
Hypsilophodon	Barremian–early Aptian	129.4	119	124.2	124	(Norman et al., 2004)
Stegoceras	late Campanian	76.5	75	75.75	76	(Arbour et al., 2009)
Archaeoceratops	Albian	112.95	100.5	106.725	107	
Asiaceratops	early Cenomanian	100.5	96.24	98.37	98	
Bagaceratops	Campanian	83.64	72.05	77.845	78	
Centrosaurus	late Campanian	76.4	70.6	73.5	74	
Leptoceratops	late Maastrichtian	68	66.04	67.02	67	
Graciliceratops	Cenomanian – Santonian	100.5	83.64	92.07	92	
Montanaceratops	early Maastrichtian	69.4	69.2	69.3	69	
Protoceratops	late Campanian	76.38	72.05	74.215	74	
Triceratops	late Maastrichtian	68	66.04	67.02	67	
Udanoceratops	late Campanian	76.38	72.05	74.215	74	
Zuniceratops	mid- to late Turonian	92.9	89.77	91.335	91	
Yamaceratops	Santonian – Campanian	86.26	72.05	79.155	79	
Prenoceratops	late Campanian	75.0	75.0	75.0	75	
Cerasinops	early Campanian	80	76.5	78.25	78	
Zhuchengceratops	?late Campanian – Maastrichtian	76.38	66.04	71.21	71	
Auroraceratops	Albian	112.95	100.5	106.725	107	
Mosaiceratops		92	77	84.5	85	
Liaoceratops	late Barremian – early Aptian	125	122	123.5	124	(Smith et al., 1995)
Psittacosaurus mongoliensis	Aptian / Albian	126.3	100.5	113.4	113	

Taxon	Age	Lower Bound	Upper Bound	Midpoint	Date Assigned	Source
Psittacosaurus sinensis	Aptian / Albian	126.3	100.5	113.4	113	(Qiu et al., 2001)
Chaoyangsaurus	Tithonian	152.1	145	148.55	149	(Zhao et al., 1999)
Xuanhuaceratops	Late Jurassic	152.57	145.01	148.79	149	
Yinlong	Oxfordian	163.47	157.25	160.36	160	
Aquilops	middle – late Albian	108.5	104	106.25	106	(Farke et al., 2014)

References

Arbour, V. M., M. E. Burns, and R. L. Sissons. 2009. A redescription of the ankylosaurid dinosaur *Dyoplosaurus acutosquameus* Parks, 1924 (Ornithischia: Ankylosauria) and a revision of the Genus. Journal of Vertebrate Paleontology 29:1117-1135.

Chinnery, B. J., and J. R. Horner. 2007. A new neoceratopsian dinosaur linking North American and Asian taxa. Journal of Vertebrate Paleontology 27:625-641.

Farke, A. A., W. D. Maxwell, R. L. Cifelli, and M. J. Wedel. 2014. A ceratopsian dinosaur from the Lower Cretaceous of Western North America, and the Biogeography of Neoceratopsia. PLoS One 9:e112055.

Han, F. 2013. The Osteology of *Yinlong downsi* (Ornithischia: Ceratopsia) and the Phylogeny of the ornithischian dinosaurs. PhD Dissertation thesis/dissertation, University of Chinese Academy of Sciences, Institute of Vertebrate Paleontology and Paleoanthropology, Chinese Academy of Sciences, 388 pp.

Makovicky, P. J. 2010. A redescription of the *Montanoceratops cerorhynchus* holotype with a review of referred material; pp. 68-82 in M. J. Ryan, B. J. Chinnery-Allgeier, and D. A. Eberth (eds.), New perspectives on horned dinosaurs: The Royal Tyrrell Museum Ceratopsian Symposium. Indiana University Press, Bloomington.

Makovicky, P. J., and M. A. Norell. 2006. *Yamaceratops Dorngobiensis*, a new primitive ceratopsian (Dinosauria: Ornithischia) from the Cretaceous of Mongolia. American Museum Novitates:1-42.

Morschhauser, E. M. 2012. The anatomy and phylogeny of *Auroraceratops* (Ornithischia: Ceratopsia) from the Yujingzi Basin of Gansu Province, China. PhD Dissertation thesis/dissertation, University of Pennsylvania, 629 pp.

Norman, D. B., H.-D. Sues, L. M. Winter, and R. Coria. 2004. Basal Ornithopoda; pp. 393-412 in D. B. Weishampel, P. Dodson, and H. Osmólska (eds.), The Dinosauria. The University of California Press, Berkeley, CA.

Pol, D., and M. A. Norell. 2001. Comments on the Manhattan Stratigraphic Measure. Cladistics 17:285-289.

Qiu, J., D. Wang, Q. Luo, and H. Liu. 2001. ^{40}Ar-^{39}Ar dating for volcanic rocks of Qingshan Formation in Jiaolai Basin, Eastern Shandong Province: A case study of the fenlingshan volcanic apparatus in Wulian County. Geological Journal of China Universities 7:351-355.

Ryan, M. J., D. C. Evans, P. J. Currie, C. M. Brown, and D. Brinkman. 2012. New leptoceratopsids from the Upper Cretaceous of Alberta, Canada. Cretaceous Research 35:69-80.

Sereno, P. C. 2000. The fossil record, systematics and evolution of pachycephalosaurs and ceratopsians from Asia; pp. 480-516 in M. J. Benton, M. A. Shishkin, D. M. Unwin, and E. N. Kurochkin (eds.), The Age of Dinosaurs in Russia and Mongolia. Cambridge University Press, Cambridge.

Smith, P. E., N. M. Evensen, D. York, M.-M. Chang, F. Jin, J.-L. Li, S. Cumbaa, and D. Russell. 1995. Dates and rates in ancient lakes: ^{40}Ar–^{39}Ar evidence for an Early Cretaceous age for the Jehol Group, northeast China. Canadian Journal of Earth Sciences 32:1426-1431.

Xu, X., C. A. Forster, J. M. Clark, and J. Mo. 2006. A basal ceratopsian with transitional features from the Late Jurassic of northwestern China. Proceedings of the Royal Society B: Biological Sciences 273:2135-2140.

You, H.-L., and P. Dodson. 2003. Redescription of neoceratopsian dinosaur *Archaeoceratops* and early evolution of Neoceratopsia. Acta Palaeontologica Polonica 48:261-272.

Zhao, X.-J., Z.-W. Cheng, and X. Xu. 1999. The earliest ceratopsian from the Tuchengzi Formation of Liaoning, China. Journal of Vertebrate Paleontology 19:681 - 691.
